# Supplementary material for: Tuberculosis in Migrant Populations. A Systematic Review of the Qualitative Literature
Source: PLoS One. 2013 Dec 5;8(12):e82440. doi: 10.1371/journal.pone.0082440 (PMC3857814; doi:10.1371/journal.pone.0082440)
Supplement: Checklist S1 — PRISMA checklist. (DOCX) [file pone.0082440.s001.docx]

Tuberculosis in migrant populations. A systematic review of the qualitative literature.

Bruno Abarca Tomás^1^*, Christopher Pell^2,3^, Aurora Bueno Cavanillas^1,4,5^, José Guillén Solvas^1,4,5^, Robert Pool^2,3^, María Roura^2^

1 Department of Preventive Medicine and Public Health, University of Granada, Granada, Spain.

2 Centre for International Health Research (CRESIB), Hospital Clínic-Universitat de Barcelona, Barcelona, Spain.

3 Centre for Social Science and Global Health, University of Amsterdam, Amsterdam, The Netherlands.

4 Service of Preventive Medicine, San Cecilio University Hospital, Granada, Spain.

5 Consorcio de Investigación Biomédica en Red en Epidemiología y Salud Públic, (CIBERESP), Madrid, Spain

# * E-mail: brunoat@correo.ugr.es Abstract:

## Background

The re-emergence of tuberculosis (TB) in low-incidence countries and its disproportionate burden on immigrants is a public health concern posing specific social and ethical challenges. This review explores perceptions, knowledge, attitudes and treatment adherence behaviour relating to TB and their social implications as reported in the qualitative literature.

## Methods

## Systematic review in four electronic databases. Findings from thirty selected studies extracted, tabulated, compared and synthesized.

## Findings

TB was attributed to many non-exclusive causes including air-born transmission of bacteria, genetics, malnutrition, excessive work, irresponsible lifestyles, casual contact with infected persons or objects; and exposure to low temperatures, dirt, stress and witchcraft. Perceived as curable but potentially lethal and highly contagious, there was confusion around a condition surrounded by fears. A range of economic, legislative, cultural, social and health system barriers could delay treatment seeking. Fears of deportation and having contacts traced could prevent individuals from seeking medical assistance. Once on treatment, family support and *“the personal touch”* of health providers emerged as key factors facilitating adherence. The concept of latent infection was difficult to comprehend and while TB screening was often seen as a socially responsible act, it could be perceived as discriminatory*.* Immigration and the infectiousness of TB mutually reinforced each another exacerbating stigma. This was further aggravated by indirect costs such as losing a job, being evicted by a landlord or not being able to attend school.

## Conclusions

Understanding immigrants’ views of TB and the obstacles that they face when accessing the health system and adhering to a treatment programme-taking into consideration their previous experiences at countries of origin as well as the social, economic and legislative context in which they live at host countries- has an important role and should be considered in the design, evaluation and adaptation of programmes.

# Introduction

Declared a global health emergency by the World Health Organization (WHO) in 1993, tuberculosis (TB) remains an important cause of mortality and morbidity worldwide. In spite of the gradual decline during the 1990s and 2000s, there were around nine million incident cases of TB in 2010, which translates into a global incidence of 128 per 100,000. In the same year, 1.4 million persons died as a result of the disease [1].

Caused by Mycobacterium tuberculosis, TB is an air-borne disease transmitted when a person with the active pulmonary disease coughs, sneezes, speaks or spits. The main symptoms are a persistent cough (sometimes producing blood), fever, sweating, weakness and weight loss. In some cases, TB can affect other organs. Although it is estimated that one third of the world’s population might be infected with TB, not all infected individuals develop the disease. For many years, the infection can remain latent, asymptomatic and non-infectious. However, the infection can reactivate at any time and this occurs in up to 10% of cases.

Clinically suspected TB is mainly confirmed with a chest X-ray and the identification of the bacterium in blood/sputum samples. A cutaneous Tuberculin test can also be used for diagnose [2]. Treatment involves combinations of antibiotics that are determined by the resistance characteristics of the infection. Several months of treatment are required to cure TB, but after two weeks the likelihood of transmission reduces [3]. However, treatment adherence is often low [4]. In terms of prevention, the BCG (Bacillus Calmette-Guérin) vaccine is effective during childhood and is carried out systematically in many countries [5]. However, the published research presents no evidence of a protective effect ten years after vaccination [6].

The re-emergence of TB in low-prevalence industrialized countries is a public health challenge. Multiple antibiotic resistance and TB’s interaction with HIV/AIDS are however only part of this [2]; TB is strongly linked to poverty, both in less-developed countries, where 95% of the deaths linked to the disease take place, and pockets of poverty and marginalization in developed countries. Ethnic minorities, the homeless, injecting drug users, prisoners and migrants – economic and labour migrants, asylum-seekers, refugees, documented or undocumented – are population groups whose vulnerability to the disease is increased and their access to health services complicated by social discrimination, isolation and poverty [3].

Multiple socio-economic, cultural, environment and political factors underpin the recent increases in human migration that have contributed to changes in patterns of TB infection [4]. Indeed, recently, many Western countries have experienced increased rates of TB infection. In North America, Europe and Australasia, the disease disproportionally affects foreign-born populations from Africa, Asia and/or Latin America, where TB infection rates are much higher. In low-incidence countries, the rate of infection amongst foreign-born migrants reaches ten or twenty times that of the autochthonous population, and represents 60% to 70% of the recorded cases. Nonetheless, infection rates vary within the immigrant and non-immigrant population: although various studies report low infection rates in the non-immigrant population [5] and that transmission outside of small closed community or nuclear families is exceptional, some recent research highlights possible instances of transmission between distinct population groups [6–8]. The risk of transmission within the immigrant communities in the host country is however without question.

In industrialized low-incidence countries with high levels of immigration from less-developed countries, the basic TB control strategy consists of minimizing transmission through identifying and curing a high proportion of cases (above all bacteriologically confirmed cases). The fundamental elements of this strategy are: a) early TB detection, mainly in most vulnerable groups; b) adequate treatment adherence for active infections; and c) in certain circumstances, detection and prophylactic treatment of latent asymptomatic infections [9–11].

There are notable differences between countries with regard to their TB screening programmes for immigrants, in terms of screening location, the programmes’ administrative and financial autonomy and the procedures followed. In general, because of the ease with which at-risk groups can be reached, migrants are screened on arrival or during the processing of temporary residence applications in the host country. In some instances, for diagnosis and to complete treatment over several months, referrals to specialized clinics are given. However, patients can be repatriated or deported prior to completing treatment. Many treatment programmes involve Directly Observed Treatment (DOT) policies, by specially trained health personal, which seeks to ensure that patients follow the necessary treatment regimen.

Although often unnoticed, reactivation of latent infection is common amongst immigrants and is responsible for many of the TB cases that occur up to several years after arrival in the host country [12]. An asymptomatic infection cannot be detected by the patient or by chest X-ray and the tuberculin skin test produces many false-negatives (as a result of immunosuppression or incorrect administration) and false-positives (as a result of immunisation). TB’s complex symptomology also interferes with accurate diagnosis. Therefore, opportunistic interventions are not enough to detect latent infections and active surveillance is required [13]. Screening individuals who have been in contact with TB is however difficult: identification and tracing is challenging, and diagnosis requires at least two visits to a health centre.

In general, screening for latent TB is only cost-effective amongst particularly vulnerable groups, such as immuno-compromised individuals or those who have had recent contact with an active TB infection [14]. In spite of the high proportion of cases amongst immigrants in low-incidence countries, there is debate about the screening programmes and their public health impact [15,16].

Migrants’ social, legal and economic circumstances can have a detrimental effect on TB’s disease progression, its diagnosis and treatment. However, TB can also exacerbate many of these factors. Although migration is a highly heterogeneous process, it is often highly stressful [17] and the challenges that migrants face – communication problems, loss of social support, adapting to new surroundings, discrimination, acculturation – can be aggravated by fear of TB, the stigma linked to a positive diagnosis or the changes in life-style related to TB treatment [4,18].

Currently, TB control and treatment programmes in low-incidence countries that host large numbers of migrants face challenges brought about by changing patterns of TB. The general aim of this review is to explore immigrants’ perceptions of TB and TB control programmes. Understanding these perceptions has an important role to play in evaluating and adapting current programmes. Specific objectives include: a) to examine their knowledge of, attitudes towards and beliefs about TB; b) to analyze factors related to seeking TB care; c) to analyze factors influencing treatment adherence; and d) to describe the social repercussions of a positive TB diagnosis.

# Methods

## Systematic review

A systematic review of qualitative studies was carried out following a meta-ethnographic approach [19]. Using rigorous methods and analysis, systematic reviews seek to identify, evaluate and synthesize the scientific evidence from primary studies in a specific research area. Qualitative research is necessary for the in-depth analysis of knowledge, attitudes and experiences. Taking a broad perspective, the combination of both approaches – systematic reviews and qualitative research – is relevant to the study of immigrants’ views and attitudes towards TB. The use of this approach complements quantitative research by offering detailed explanations of and insights into the relationships between different variables [20].

## Search strategy

Searches were carried out in the following databases: MEDLINE, SCOPUS, JSTOR and EMBASE. Search topics included “Tuberculosis”, “immigrants” and “qualitative research” using the search terms and MeSH descriptions described in Table 1. For the concept “immigrant”, a broad interpretation was used, including rural to urban migration, asylum-seekers and refugees. The search and selection was carried out in October and November 2011. Two of the papers reviewed [21,22] were included as electronic publications and have since been fully published. Studies that address migration and TB, based on the opinions and experiences of migrants, health professionals and other key informants were included.

A hand search was also undertaken of the bibliographies of the selected articles. A final internet search was conducted using Google Scholar, in order to minimize the potential loss of relevant sources. The search and article screening process was reviewed by a second researcher.

## Inclusion criteria

We included qualitative studies, as well as studies that combined both qualitative and quantitative methods in which qualitative results were reported and described clearly. The search was limited to studies in English, French or Spanish. There were no limits regarding place of study or publication date.

## Study selection

The initial search identified 3,150 articles, which were reduced to 2,501 after eliminating duplicates. After reviewing titles and abstracts 2,301 more were excluded as irrelevant for the research topic. Of the 200 remaining, by reading the abstracts and the full text versions, 23 articles were selected as meeting inclusion criteria. Reviewing the bibliographies of these articles and the additional Google Scholar search identified seven further articles that met inclusion criteria [22–28]. A total of 30 articles were finally included for review (Figure 1).

## Categories of analysis

Four categories were developed for the analysis of findings: a) knowledge of, attitudes towards and beliefs around TB; b) seeking healthcare and TB diagnosis; c) TB treatment and prophylaxis; and d) social repercussions of TB.

# Results

## The studies and study participants

Published between 1997 and 2011, as Table 2 shows, the included studies were varied in terms of research methods, participants and study site. In-depth individual interviews was the method most commonly used (24 studies), followed by focus groups (12 studies), participant observation (5 studies), case studies (1 study) and other methodologies (6 studies). Several studies combined qualitative methods or used them together with quantitative methods. Participants included patients receiving treatment, healthy persons with latent TB, untreated TB patients, health professionals and other key informants. Generally, respondents were migrants from rural to urban contexts or from high to low TB incidence countries.

## Knowledge of, attitudes towards and beliefs about TB

Various studies showed a generally low level of knowledge about TB and widespread misconceptions about TB transmission. Some respondents had basic notions of bacteria and infection with regard to air-borne transmission [25,29–31] but participants in other studies attributed the disease to excessive labour [32], irresponsible lifestyles [24,32], genetic inheritance, poisoning, pneumonia [33], weather/climate conditions [25], weakness caused by physical exhaustion, malnutrition or living conditions [4,22] as well as exposure to chemical products [30], dirt, exposure to low temperatures [26,31], stress and witchcraft [34]. Somali women, for example, considered TB to be a divine punishment for previous “dishonesty” [21]. Vietnamese immigrants in the US differentiated “psychological TB”, caused by physical or environmental conditions, from “physical TB” to which psychological TB could progress following bodily deterioration and weakness of the immune system [35].

For many respondents, the modes of TB transmission, or preventive methods were unclear, and different explanations of disease aetiology sometimes co-existed [36]. The belief that TB is transmissible through contact with contaminated objects or utensils or through brief direct contact with a TB sufferer was common [21,23,28–30,33]. Although in one study the majority of the respondents did not believe that TB was contagious [37], in general, amongst migrants, TB was perceived to be highly contagious [23,25,30,33].

*“ (You can get TB) just being around someone who has it, or drinking out of their glass, or eating off of their plate, or by having relations with a woman who has TB”*

Mexican migrant farmworker in the US [30].

Thought of as an important but treatable disease [25,29] that might be fatal, immigrants were afraid of TB’s severity [23,24,30,31,33,38,39]. Others perceived that although TB was a serious disease in their countries of origin the disease was not equally severe in host countries [21,25,26,34,38,40]. In some cases, participants believed that they were different diseases in the country of origin and in their host country [18,41].

*“If you get TB, you die.”*

Male Mexican immigrant in the US, with latent TB [38].

*“Nobody knows, except my family… But they (the doctors) said that it isn’t like the TB that we used to have that was very contagious and very bad.”*

Female Samoan TB patient in New Zealand [41].

*“...I don’t think Somalis here, in America, have it. Maybe people in Africa...”*

Male Somali immigrant in the US [21].

Tuberculosis was mainly perceived as a disease of the lungs, with respiratory symptoms including cough and coughing up blood, but also non-respiratory symptoms like fever, weight loss and general tiredness [22,23,25,28,30,35]. It was uncommon for respondents to know that TB had other non-pulmonary effects [26,34] and many of the initial symptoms were not noticed or attributed to other conditions [33,40].

*“People were mixing TB up with lung cancer, asthma, so the term, particularly the formal term of TB is not very well understood. It’s sort of lumped in with other lung conditions... There is a lot of miscommunication within the community. Not only some fears about this, but a whole lot of muddle around the condition.”*

Male Chinese immigrant in Canada [37].

Many study participants had never heard of latent TB before being diagnosed [22,25,38] and, as the relationship between infection and disease was confusing, for many respondents it was a condition difficult to comprehend as “a disease” [18].

*“Latent TB has no symptoms and the one who has cannot infect others, then why call it a disease?”*

Male Somali immigrant in Sweden [18].

In other cases, the positive result of a Tuberculin skin test was perceived as a very serious clinical diagnosis [39].

*“When my son was diagnosed with latent TB infection, I thought he was going to die.”*

Male Mexican immigrant in the US [38].

Respondents generally understood that the vaccination helped to prevent TB [31], though in some cases there was a false sense of over-protection [29,30,38] and the distinction between BCG vaccination and the TB test [29] was not always clear.

Many participants in numerous studies talked of how their situation as immigrants could negatively influence their health or increase their exposure to TB. A weak social network [32], illiteracy [25], adverse conditions experienced during their journeys in packed and poorly ventilated vehicles [4], temporary residence in illegal refuges or detention centres [42], difficult labour conditions for illegal immigrants and police extortion [35] were among the problems mentioned.

## Seeking healthcare and TB diagnosis

Factors encouraging immigrants to seek healthcare included the desire to receive a negative result to avoid stigma associated with suspected TB infection; the right to stay legally in the host country until completing treatment [22]; wanting to find out about ones’ health status, and willingness to protect family members from infection [22].

Barriers to accessing healthcare services and therefore TB diagnosis, treatment or prophylaxis included lack of knowledge of existing free TB diagnosis and screening services at specifically designed centres [43,44]; illiteracy or lack of familiarity with the local language [22,32,45]; fear of a painful test or the social consequences of a positive result [21,25,26]; having to miss work to attend a clinic appointment [30,46,47]; transport difficulties [21,36]; queues and waiting lists; not having health insurance; irregular residence status [22]; feeling “singled out” [29,34] and the stigma associated with being seen enter a TB clinic [34]; economic costs of medical consultations [40]; dissatisfaction or cultural differences with “Western” medical services [25,34,40,41], and the presence of “clinics for immigrants” in dangerous neighbourhoods [45].

Delays in diagnosis or seeking healthcare were common and sometimes serious ranging from days to months since the onset of symptoms [22,25,26,46]. Some studies highlighted that immigrants often initially played-down the importance of symptoms, to later self-diagnose and self-medicate, using pharmacies or private clinics and finally public healthcare centres [41,42]. The absence of symptoms despite contact with persons infected with TB led migrants to pay little attention to prevention or screening [21,27].

*“At the beginning, I thought it might just be a cold, so after three or four days, I went twice to a pharmacy to buy some drugs. But I felt I was coughing more heavily after taking the medicine, so I decided to go to see a doctor in a private clinic.”*

Rural-born female Chinese migrant who suspected TB [44].

*‘‘If they’re talking about something where it’s preventive (TB screening), you know they’re not sick, then you know, there’s little motivation to do it…’’*

Immigrant in a literacy centre in the US [21].

*“I started coughing in November and it wasn’t until mid-January that I was referred to hospital for chest X-ray.”*

Male Nigerian immigrant receiving TB treatment in the UK [33].

Experiences of diagnostic processes and settings were varied. In some cases, the norm was to pass through many health centres and see many health professionals prior to receiving a TB test, which led to high patient costs [46]. Participants attributed this to health staff’s lack of training in TB diagnosis and treatment [48], poor coordination and awareness of registration and referral procedures of suspect cases [44], especially in the case of irregular migrants whose cases imply an extra bureaucratic burden [22].

As for the screening process, it was generally well received by study participants and seen as a socially responsible act in terms of helping to prevent further cases [29]. There were, albeit less frequent, comments about the different approach to screening immigrants compared to the host population [29], a distinction that made some immigrants feel discriminated [45]. In one study with Vietnamese immigrants in the US, compliance was high because clients assumed the test had to do with their visa process [45].

*“Actually it is a good idea because if you do it (get screened for TB), you know you have it then you can cure it. If you don’t cure it you can carry on giving it to other people and then that is another problem to the country.”*

Male Ghanaian immigrant with suspected TB in the UK [29].

*“She only say me I have to take my blood pressure and my urine from me, she haven’t mentioned the tuberculosis test but when I came and she saw perhaps I am a black person, or something like that, I am not saying she is racist or something like that, I don’t say that, but I think it is because I am African.”*

Male African immigrant with suspected TB in the UK [29].

Some respondents expressed anxiety with regard to diagnostic procedures and others feared the lack of privacy, worrying particularly about the presence of non-medical administrative staff and health professionals from their own country [34].

TB control often involves collection of epidemiological data and tracing the contacts of diagnosed persons in order to administer prophylactic antibiotics. In some studies, due to fears about deportation, immigrants with irregular residence status would not be willing to reveal details of their migratory route [18,22] and the requirement to provide information about contacts could even prevent individuals from seeking medical assistance [34]. The fact that migrants might not reside with family and friends rather that at their official address further complicated the task of tracing contacts [18,41].

*“Once Smittskydds people put you in their books (records), they follow not only the patient but the whole family and this puts fear and suspicion in peoples’ minds. . . so people see themselves as “marked” and not see the advantage of controlling infection. Nevertheless, they feel pursued by authority.”*

Male Somali immigrant, ill with TB in Sweden [18].

## TB treatment and prophylaxis

Problems of accessibility can constitute a barrier to health seeking and early diagnosis, and can also present obstacles to prophylaxis and the periodic visits that adequate TB treatment necessitates. At times, the need to travel [40], difficulties in understanding complex information in a strange language [40,45] or in a way considered too mechanical and impersonal [45], lack of awareness of free treatment [22] or the form in which and the rigid opening hours when medication is provided do not correspond with patients’ working hours and lifestyles [32,36,47]. The use of interpreters as part of healthcare for TB patients and during their periodic visits was also problematic – due to the sensitivity of the information and the fear of loss of privacy and stigmatization [18].

Factors facilitating adherence to treatment included family support [45], receiving personal advice from health staff and social contacts and receiving care provided by staff specially trained in TB [33] or with sensitivity and the ability to establish a personal relation on the same cultural terms [43], like someone who *“takes you from far and brings you in close”*, as described by Haitian migrants [34]. Having positive relationships with health professionals was perceived to be a crucial element [38] especially when close contact was established through home visits and phone discussions [46].

*“We do know that tuberculosis is not difficult to treat. The problem is that health care services have to give the medication but they have no control if people will take it. How do we deal with this problem? You must have a strategic plan. First, the education is too mechanical... you need the personal touch.”*

Male Vietnamese community leader and immigrant in the US [45].

The length of TB treatment and prophylaxis caused changes in migrants’ lives [24] and this sometimes hampered adherence [34]. Participants often reported “controlling their treatment” and taking their medication, for as long as necessary, on their own, with follow-up from health professionals or with the support of relatives [46,47]. However, some faced problems, when they had to periodically attend the health centres where they received DOT [32]. The absence of symptoms could equally lead to poor adherence and treatment failure [40]. In spite of health professionals’ explanations, without symptoms, some patients questioned the need to continue treatment [39] and gave a false impression of adherence. In a study conducted in the United States, both the director of a clinic and migrant community leaders were worried about TB patients’ non-adherence to TB treatment. At this site, some nurses tried to coax patients into prophylaxis by assuring them that after completing treatment they would receive a certificate that would be helpful to get a job or send their children to school. The patients came to the appointments but did not take the medication [45]. Specific health policies adopted in certain countries could also have a negative impact on treatment adherence: in Oman, TB patients were deported after successful treatment, so many immigrants went into hiding and abandoned treatment [48].

Even though in most countries TB screening, prophylaxis and treatment is free, two studies carried out in China reported problems associated with the overall costs [44,46]. This occurred because patients had to initially pay the costs or because, in addition to the antibiotic treatment, other liver medication or repeated X-rays were prescribed (in spite of the lack of supporting scientific evidence). These interventions were not free and led to complaints. Interviews with health professionals revealed that most were unaware of the policy of free TB diagnosis and treatment.

*“The free treatment policy means I need to pay all the medical costs first. It does little to release my current financial burden in treating TB.”*

Male Chinese rural-to-urban migrant, diagnosed with TB [46].

In two US studies, respondents regarded traditional remedies as complementary to modern treatments, which were considered as the most effective against TB [23,28]. However, in New Zealand, ambivalence towards the advantages of modern, as opposed to traditional therapy, could lead to poor adherence [40]. Beliefs that free-of-charge generic drugs were of a poor quality also prevented some individuals from taking the prescribed medication [21,44].

*‘‘(Treat with) not generic medicine, good medicine… Generic’s no good medicine.’’*

Somali immigrant in the US [21].

Adverse effects of TB medication could also lead to poor treatment adherence [21,37,45]. There were reports of general fatigue, fever, irritability, memory loss, nausea, constipation, loss of sense of taste, gastritis and headaches [33,39,45]. In some cases, the antibiotics were perceived as “strong” and the participants therefore turned to traditional medicines from their home countries to counter the side effects until the course of treatment was completed [4,23]. Doctors’ explanations about side effects and their symptoms, even if properly translated into the immigrants’ language, could be incongruent with the immigrants’ explanatory model of disease [45].

There was a variety of expectations about antibiotic treatment or preventive therapy for latent TB. Some studies suggested that preventive therapy would be accepted if a full explanation were given, whereas others were more sceptical [34]. In some communities, the benefits of treating latent TB were well understood [23]. This included efficacy of the prescribed medication [38], avoidance of stigma associated to exhibiting TB symptoms, and an appreciation that the risk of infecting others would be reduced [31].

*“It will kill the germs... stop the virus from continuing... the infection will disappear... I will feel better, not to be contagious especially for my children...”*

Latin American immigrant, ill with TB in the US [31].

## The social repercussions of TB

Experiences of TB-related stigmatization were reported in most of the studies. Feelings of stigmatization derived from being labelled as an “at-risk group” began within the health system when some participants perceived that health staff treated immigrants differently [34,37,43,48]. Immigration and the infectiousness of TB mutually reinforced each another, magnifying feelings of being “out of place” and exacerbating the illness experience [43].

*“...don’t splash all over the mainstream media that immigrants have TB:*

*it will just make the discrimination that might exist, even higher.”*

Immigrant and interview facilitator in Canada [37].

Feelings of stigma influenced immigrants’ attitudes towards prevention, diagnosis and treatment [24,28,34,37,41,46] and could prevent them from sharing relevant information with their doctors, including TB-related symptoms [50]. At the community level, TB was considered shameful, dirty and sinful, and a direct consequence of the “wrong behaviour” of the patient or a family member [21,23,25,27]. Attempts at hiding a TB diagnosis from other community members were thus frequently reported [21,25,30,36,38,41,46].

*“I do not want community doctors to visit me regularly. Others will know that I am sick and have TB. My landlord will expel me if he knows (I have TB).”*

Chinese rural to urban immigrant, ill with TB [46].

In a Spanish study, immigrants feared getting sick again when noticing minor symptoms or seeing someone close to them cough, which had a negative psychological effect even after they were cured [24]. The association of symptoms such as weight loss and coughing with HIV/AIDS increased the stigma linked to TB [33] as did the association of TB with drug use [25].

*“These days, if you have TB, they say it’s AIDS. If you have pneumonia, they say it’s AIDS. If you have common fever, make sure you stay inside your house! Once you lose one kilogram, you’re finished. Some won’t even shake your hands or eat with you. The stigma is too much. So people prefer to die.”*

Male Nigerian immigrant, ill with TB, in the UK [33].

Fear of being infected [32,33], anticipating stigma, shame or a negative reaction [27,34,36–38,43] and attempts to safeguard personal or family dignity [41] led individuals to hide the diagnosis from family or friends, to isolate themselves and weaken their social network even more. Eventually, they might even be rejected by family, partner, or close friends [24,28,34].

*“I remember in our family, one of our relatives had TB, and we isolated him. It used to scare the heck out of me. We would talk to him from a distance.”*

Philippine immigrant in the US [28].

With few economic resources and savings to start with, a TB diagnosis entailed a range of direct costs associated with the illness and medical procedures as well as indirect costs such as losing a job [32], being evicted by a landlord [4] or not being able to attend school [24,32,36,43,46]. The social and economic consequences of TB could sometimes lead to mental health problems [23,43].

# Discussion

When analyzing immigrants’ experiences and views of TB, many authors highlighted the influence of misconceptions about the disease, its aetiology and its prognosis. In many cases, past TB-related experiences of family members and in the country of origin – where the prevalence and severity of TB tends to be more pronounced – were the first contact with and source of information about the disease [21,35]. The many transmission routes cited in the studies were underpinned by the belief that casual contact with an infected person or objects that s/he has touched could cause infection. The association of the disease with inappropriate behaviour and the severity attributed to TB sometimes resulted in an escalation of fear and stigmatization.

Misconceptions were accentuated by contradictions in the information received from health professionals in migrants’ home and host countries [38]. In their home countries, the disease was familiar but latent infection unknown [38]. In host countries, systematic BCG vaccination was widespread and accepted, but many were unaware of the loss of immunity over time [49,50]. On arrival in the host country, faced with new complex concepts, the already-internalized explanatory model of TB had to be revised and compared with the new explanations offered. Partly depending on the success of this process, the patient would decide whether or not to adhere to treatment because they do not understand it, do not consider it necessary (because of previous vaccination, for example), or do not trust the new model.

Delayed TB diagnosis is frequent amongst migrants [21,22,25–27,33,40,41,44,46]. Undocumented immigrants in the US, for example, and urban-to-rural migrants in China, delay a few weeks with symptoms prior to seeking medical help compared with the autochthonous populations [51,52]. The factors identified in this review coincide with those found in the wider literature: language difficulties, fear of immigration authorities, concerns about costs, unemployment, and low levels of education (although its impact is not well-known). Also the difficulties in accessing the health system, poor awareness of symptoms and fear of diagnosis contribute to delayed diagnosis. Fear of immigration authorities complicates tracing patients’ contact and screening for latent infection. Moreover, immigrants tended to access other lower-quality healthcare resources, prior to attending the appropriate health centre, increasing costs and lengthening the delay [41,44].

Although there are different screening strategies, screening that is carried out with coercion or linked to entry or legalizing residence reaches more immigrants [53,54]. However, such approaches, though not always identified as discriminatory, have a questionable public health impact [53,55] and should be examined from an ethical perspective [56]. Screening on entry, (based on the assumption that country of origin is the main risk factor) excludes undocumented immigrants, who do not enter by the designated routes, and immigrants that are not ill or infected on entry. Coercion can also be counter-productive if it is accompanied by partial or insufficient information about the diagnosis, the disease or latent infection, and thus unable to provide valid arguments to migrants. Patients can ignore and doubt the clinical importance of screening if the explanation does not coincide with their views, or the symptoms do not develop as expected. When that happens, coercion can have a lesser impact and even lead to rejection. Even when screening programmes are not compulsory, these should be a component of a wider approach, rather than a stand-alone intervention [57].

## There were not many references to compulsory screening and treatment policies at borders or entry point in the literature reviewed. Acceptability of screening and opinions of TB patients identified in these settings may differ.

In spite of the fear that TB provokes, there was also a false sense protection, both amongst immigrants, who assume that they have left behind the hazardous circumstances, and the health professionals, who do not suspect TB if they do not recognize the risk of reactivation of latent TB, are not familiar with diagnostic procedures or symptoms are unclear. In market-orientated health systems, financial incentives for health professionals can increase the number of appointments and diagnostic tests carried out prior to a TB diagnosis [48].

Interactions with health professionals – initially and during the follow up that treatment requires – were highlighted as a fundamental element of treatment adherence. Cultural understanding, personal closeness and immigrants’ trust in health professionals, whether of the same background or not, was a key factor for adherence and encouraged diagnosis.

Programme design and assessment of adherence should also be reassessed. For example, the use of indicators linked to periodic collection of medication from health centres or the absence of adequate treatment surveillance, could lead to false image of success. Similarly, prescribing medication but providing partial information combined with coercion led to treatment initiation but did not guarantee long-term adherence. Moreover, although programmes of TB diagnosis and treatment might be free, the most marginalized immigrant populations often lacked awareness regarding these programmes and at times they faced other, often unnecessary, indirect costs.

The use of “traditional” remedies did not present an obvious threat to biomedical treatments, even if at times immigrants found it difficult to adopt them. In turn, traditional knowledge about diet or natural remedies could help patients to deal with common adverse effects of treatment.

Although TB-related stigma has been widely discussed elsewhere [3], it has often received more attention because of its impact on TB screening and treatment rather than as a consequence of these programmes. Fears about the illness and concerns about the reactions of others can lead to symptoms being ignored and discourage medical attention, rejection of diagnosis or poor treatment adherence.

Immigrants’ fear of TB was based on previous experiences in their home countries and was sometimes translated into community and personal marginalization weakening the patients’ closest social networks, often key to providing the long-term support required for a successful completion of TB treatment.

The social and economic consequences of TB extended beyond perceptions of stigma. The marginalization of the patient was often described, and the costs associated with the disease and the physical weakness, unemployment, or reduced employment capacity could have serious repercussions for a patient’s economic situation, social support and mental health. These problems, together with prevailing misconceptions about the disease and its prognosis, should be taken into consideration and addressed in TB treatment and control programmes.

Compared to the general population, immigrants are more vulnerable to TB and disproportionally affected by the disease [3,4,25,32,36,42,43]. However, the circumstances that present a risk are not only related to the epidemiological, social and economic conditions at immigrants’ home countries [58,59]. The circumstances of the migratory process and living conditions at host countries including social isolation, illiteracy, overcrowding, poverty, labour conditions and irregular residence status are also factors to be considered. Although a large proportion of TB cases in low-incidence countries is attributed to the reactivation of an “imported infection” [60,61] the importation of active TB is only a minor part of the total TB burden [62]. It has been argued that, social exclusion and poverty in the host countries can be an additional and more important factor than those related to the conditions in home countries [63,64]. The risk of TB amongst immigrants is likely to vary not only according to their country of origin, but also in relation to their reasons for migration, economic situation, legislative status, and overall living conditions.

## Limitations of the review

This review is limited by the difficulty of synthesising and comparing results of studies conducted in a wide diversity of contexts, with a variety of respondents and using different approaches. Not all studies included easily-comparable information on relevant issues, such as migrants’ legal and socio-economic economic circumstances, religion, or length of residence in the host country. The lack of information in many studies regarding the sex and age of study participants did not allow us to disaggregate the analysis by these variables despite its possible relevance. On the other hand, strengths of the review included a systematic search in four databases, supplemented by a hand search of the bibliography in the articles identified and an additional search in Google Scholar. Having no limits to the inclusion of articles regarding place of study or publication date also allowed for a broader perspective, and a comprehensive analysis of immigrants’ perceptions of TB and TB control programmes.

# Conclusions

The results of this review indicate that immigrants’ knowledge of and attitudes towards TB are largely built on their previous experiences. Even though the immigrant populations were heterogeneous, there were common challenges amongst groups, such as the perception of TB as being highly contagious and severe, frequent lack of treatment adherence, obstacles to effective communication with health providers and/or anticipated and enacted stigma that hindered treatment and isolated the patient.

However, these similarities should not obscure the needs of specific groups – defined in terms of cultural references and socio-economic factors including social networks and living conditions at host countries. Immigrants require appropriate information on TB aetiology and transmission tailored to different language abilities, levels of knowledge and beliefs systems, in addition to screening strategies and health care provision that are adapted to their particular traditions, values and social relationships, in order to guarantee information, screening, diagnosis and adherence to treatment.

Beyond escalating current interventions and increasing monitoring of TB incidence and prevalence in immigrant populations it is crucial to understand immigrants’ perceptions of TB and the specific obstacles that they face when accessing the health system, seeking a diagnosis and adhering to a treatment programme.

# Acknowledgments

Authors thank the COHEMI project study group that includes: Maurizio Bonati, Francesca Severino, Valeria Confalonieri, Chiara Pandolfini, Zeno Bisoffi, Dora Buonfrate, Andrea Angheben, Marco Albonico, Alessandro Bartoloni, Marianne Strohmeyer, Lorenzo Zammarchi, Jose Muñoz, Robert Pool, Ana Requena Mendez, Maria Roura, Joaquim Gascón, Mª Jesús Pinazo, Mª Elizabeth Posada, Laia Ventura Garcia, Anita Hardon, Christopher Pell, Peter L. Chiodini, Juan Moreira, Roberto Sempértegui, Mariella Anselmi, Eduardo Gotuzzo, Maria Alejandra Mena, Hector H. Garcia, Javier Bustos, Saul Santiva, Faustino Torrico, Daniel Lozano, Guido Chumiray Rojas, Teresa Hinojosa Cabrera, Javier Ochoa Morón, Ignacio Abapori, Cuellar, Jaime Amorós Suarez, Gianni Tognoni, Alessandra Nicoletti, Elisa Bruno. Peter L. Chiodini is also supported by the UCL Hospitals Comprehensive Biomedical Research Centre Infection Theme.

# References

1. World Health Organization (WHO) (2011) Global Tuberculosis Control 2011. Geneva: WHO.

2. Porter JD, McAdam KP (1994) The re-emergence of tuberculosis. Annu Rev Public Health 15: 303-23.

3. World Health Organization (WHO) (2005) Addressing poverty in TB control, options for national TB control programmes. Geneva: WHO.

4. Ho MJ (2004) Sociocultural aspects of tuberculosis: a literature review and a case study of immigrant tuberculosis. Soc Sci Med 59 (4): 753-62.

5. Barniol J, Niemann S, Louis VR, Brodhun B, Dreweck C, et al. (2009) Transmission dynamics of pulmonary tuberculosis between autochthonous and immigrant sub-populations. BMC Infect Dis 9: 197.

6. Littleton J, Park J, Thornley C, Anderson A, Lawrence J (2008) Migrants and tuberculosis: analysing epidemiological data with ethnography. Aust N Z J Public Health 32: 142-9.

7. MacPhrson DW, Gushulak BD (2006) Balancing prevention and screening among international migrants with tuberculosis: Population mobility as the major epidemiological influence in low-incidente nations. Public Health 120: 712-23.

8. Borrell S, Español M, Orcau A, Tudó G, March F, et al. (2010) Tuberculosis transmission patterns among Spanish-born and foreign-born populations in the city of Barcelona. Clin Microbiol Infect 16: 568-74.

9. New Zealand Ministry of Health (2010) Guidelines for Tuberculosis Control in New Zealand 2010. Wellington: Ministry of Health.

10. Schneider DL, Lobato MN (2007) Tuberculosis control among people in U.S. Immigration and Customs Enforcement custody. Am J Prev Med 33 (1): 9-14.

11. Broekmans JF, Migliori GB, Rieder HL, Lees J, Ruutu P, et al. (2002) European framework for tuberculosis control and elimination in countries with a low incidence. Recommendations of the World Health Organization (WHO), International Union Against Tuberculosis and Lung Disease (IUATLD) and Royal Netherlands Tuberculosis Association (KNCV) Working Group. Eur Respir J 19 (4): 765-75.

12. Mguire H, Dale JW, McHugh TD, Butcher PD, Gillespie SH, et al (2002) Molecular epidemiology of tuberculosis in London 1995-7 showing low rate of active transmission. Thorax 57: 617-622.

13. Moore-Gillon J, Davies PD, Ormerod LP (2010) Rethinking TB screening: politics, practicalities and the press. Thorax 65 (8): 663-5.

14. Centers for Disease Control and Prevention (2000) Targeted tuberculin testing and treatment of latent tuberculosis infection. MMWR Recomm Rep 49(RR-6): 1–51.

15. Dasgupta K, Menzies D (2005) Cost-effectiveness of tuberculosis control strategies among immigrants and refugees. Eur Respir J 25 (6): 1107-16.

16. Schwartzman K, Menzies D (2000) Tuberculosis screening of immigrants to low-prevalence countries. A cost-effectiveness analysis. Am J Respir Crit Care Med 161 (3 Pt 1): 780-9.

17. Bhugra D (2004) Migration and mental health. Acta Psychiatr Scand 109 (4): 243-58.

18. Kulane A, Ahlberg BM, Berggren I (2010) “It is more than the issue of taking tablets”: The interplay between migration policies and TB control in Sweden. Health Policy 91 (1): 26-31.

19. Atkins S, Lewin S, Smith H, Engel M, Fretheim A, et al (2008) Conducting a meta-ethnography of qualitative literature: Lessons learnt. BMC Medical Research Methodology 8: 21.

20. Hemingway P, Brereton N (2009) What is a systematic review? 2º ed. Hayward Medical Communications.

21. Wieland ML, Weis JA, Yawn BP, Sullivan SM, Millington KL, et al. (2012) Perceptions of Tuberculosis Among Immigrants and Refugees at an Adult Education Center: A Community-Based Participatory Research Approach. J Immigr Minor Health 14 (1): 14-22.

22. Huffman SA, Veen J, Hennink MM, McFarland DA (2012) Exploitation, vulnerability to tuberculosis and access to treatment among Uzbek labor migrants in Kazakhstan. Soc Sci Med 74(6): 864-72.

23. AhChing LP, Sapolu M, Samifua M, Yamada S (2001) Attitudes regarding tuberculosis among Samoans. Pac Health Dialog 8 (1): 15-19.

24. Blasco T (2010) Calidad de vida percibida por la población inmigrante con tuberculosis en la comunidad de Madrid (Quality of life perceived by the migrant population with TB in Madrid). Valladolid: Spanish Association of Sociology.

25. Johnson A (2006) Beliefs and barriers related to understanding TB amongst vulnerable groups in South East London. Health Protection Agency.

26. Sagbakken M, Bjune GA, Frich JC (2010) Experiences of being diagnosed with tuberculosis among immigrants in Norway - factors associated with diagnostic delay: A qualitative study. Scand J Public Health 38 (3): 283-90.

27. Tardin A, Dominicé DM, Ninet B, Janssens JP (2009) Tuberculosis cluster in an immigrant community: case identification issues and a transcultural perspective. Trop Med Int Health 14 (9): 995-1002.

28. Yamada S, Caballero J, Matsunaga DS, Agustin G, Magana M (1999) Attitudes regarding tuberculosis in immigrants from the Philippines to the United States. Fam Med 31(7): 477-82.

29. Brewin P, Jones A, Kelly M, McDonald M, Beasley E, et al (2006) Is screening for tuberculosis acceptable to immigrants? A qualitative study. J Public Health 28 (3): 253-60.

30. Poss JE (1998) The meanings of tuberculosis for Mexican migrant farmworkers in the United States. Soc Sci Med 47 (2): 195-202.

31. Ailinger RL, Dear MR (1997) Latino immigrants’ explanatory models of tuberculosis infection. Qual Health Res 7 (4): 521-31.

32. Kirwan DE, Nicholson BD, Baral SC, Newell JN (2009) The social reality of migrant men with tuberculosis in Kathmandu: implications for DOT in practice. Trop Med Int Health 14: 1442-7.

33. Nnoaham KE, Pool R, Bothamley G, Grant AD (2006) Perceptions and experiences of tuberculosis among African patients attending a tuberculosis clinic in London. Int J Tuberc Lung Dis 10 (9): 1013-7.

34. Coreil J, Lauzardo M, Heurtelou M (2004) Cultural feasibility assessment of tuberculosis prevention among persons of Haitian origin in South Florida. J Immigr Health 6 (2): 63-9.

35. Houston HR, Harada N, Makinodan T (2002) Development of a culturally sensitive educational intervention program to reduce the high incidence of tuberculosis among foreign-born Vietnamese. Ethn Health 7 (4): 255-65.

36. Joseph HA, Waldman K, Rawls C, Wilce M, Shrestha-Kuwahara R (2008) TB perspectives among a sample of Mexicans in the United States: results from an ethnographic study. J Immigr Minor Health 10 (2): 177-85.

37. Gibson N, Cave A, Doering D, Ortiz L, Harms P (2005) Socio-cultural factors influencing prevention and treatment of tuberculosis in immigrant and Aboriginal communities in Canada. Soc Sci Med 61 (5): 931-42.

38. McEwen MM (2005) Mexican immigrants' explanatory model of latent tuberculosis infection. J Transcult Nurs 16 (4): 347-55.

39. McEwen MM, Boyle J (2007) Resistance, health, and latent tuberculosis infection: Mexican immigrants at the U.S.-Mexico border. Res Theory Nurs Pract 21 (3): 185-97.

40. Van der Oest C, Chenhall R, Hood D, Kelly P (2005) Talking about TB: multicultural diversity and tuberculosis services in Waikato, New Zealand. N Z Med J 118: 1216.

41. Ng Shiu R, Park J, Kearns R (2008) Placing the experience of Pacific peoples living with tuberculosis in Auckland, New Zealand. In: Littleton J, Park J, Herring A, Farmer T, editors. Multiplying and Dividing: TB in Canada and Aotearoa New Zealand. Auckland: Anthropology Department, University of Auckland pp. 218-31.

42. Ho MJ (2003) Migratory journeys and tuberculosis risk. Med Anthropol Q 17 (4): 442-58.

43. Bender A, Andrews G, Peter E (2010) Displacement and tuberculosis: recognition in nursing care. Health Place 16 (6): 1069-76.

44. Long Q, Li Y, Wang Y, Yue Y, Tang C, et al. (2008) Barriers to accessing TB diagnosis for rural-to-urban migrants with chronic cough in Chongqing, China: a mixed methods study. BMC Health Serv Res 8: 202.

45. Ito KL (1999) Health culture and the clinical encounter: Vietnamese refugees' responses to preventive drug treatment of inactive tuberculosis. Med Anthropol Q 13 (3): 338-64.

46. Wei X, Chen J, Chen P, Newell JN, Li H, et al. (2009) Barriers to TB care for rural-to-urban migrant TB patients in Shanghai: a qualitative study. Trop Med Int Health 14: 754-60.

47. Wyss LL, Alderman MK (2006) Using theory to interpret beliefs in migrants diagnosed with latent TB. Online J Issues Nurs 12 (1): 7.

48. Al-Maniri A, Fochsen G, Al-Rawas O, De Costa A (2010) Immigrants and health system challenges to TB control in Oman. BMC Health Serv Res 10: 210.

49. Colditz GA, Brewer TF, Berkey CS, Wilson ME, Burdick E, et al. (1994) Efficacy of BCG vaccine in the prevention of tuberculosis: meta-analysis of the published literature. JAMA 271 (9): 698-702.

50. Sterne JA, Rodrigues LC, Guedes IN (1998) Does the efficacy of BCG decline with time since vaccination?. Int J Tuberc Lung Dis 2(3): 200-7.

51. Achkar JM, Sherpa T, Cohen HW, Holzman RS (2008) Differences in clinical presentation among persons with pulmonary tuberculosis: a comparison of documented and undocumented foreign-born versus US-born persons. Clin Infect Dis 48: 1277-83.

52. Wang W, Jiang Q, Abdullah AS, Xu B (2007) Barriers in accessing to tuberculosis care among non-residents in Shanghai: a descriptive study of delays in diagnosis. Eur J Public Health 17 (5): 419-23.

53. Klinkenberg E, Manissero D, Semenza JC, Verver S (2009) Migrant tuberculosis screening in the EU/EEA: yield, coverage and limitations. Eur Resp J 34: 1180-9.

54. Cocker R, Bell A, Pitman R, Zellweger JP, Heldal E, et al (2006) Tuberculosis screening in migrants in selected European countries shows wide disparities. Eur Respir J 27: 801-7.

55. Hardie RM, Watson JM (1993) Screening migrants at risk of tuberculosis. BMJ 307 (6918): 1539-40.

56. Coker R (2004) Compulsory screening of immigrants for tuberculosis and HIV. BMJ 328 (7435): 298-300.

57. Klinkenberg E, Manissero D, Semenza JC, Verver S. Migrant tuberculosis screening in the EU/EEA: yield, coverage and limitations. Eur Respir J. 2009; 34 (5): 1180-9.

58. Maguire H, Dale JW, McHugh TD, Butcher PD, Gillespie SH, et al. (2002) Molecular epidemiology of tuberculosis in London 1995-7 showing low rate of active transmission. Thorax 57: 617-22.

59. Ponticiello A, Sturkenboom MC, Simonetti A, Ortolani R, Malerba M, et al. (2005) Deprivation, immigration and tuberculosis incidence in Naples, 1996–2000. Eur J Epidemiol 20: 729-34.

60. Gilbert RL, Antoine D, French CE, Abubakar I, Watson JM, et al. (2009) The impact of immigration on tuberculosis rates in the United Kingdom compared with other European countries. Int J Tuberc Lung Dis 13 (5): 645-651.

61. Zuber PL, McKenna MT, Binkin NJ, Onorato IM, Castro KG (1997) Long-term risk of tuberculosis among foreign-born persons in the United States. JAMA 278 (4): 304-7.

62. Littleton K, Park J, Thornley C, Anderson A, Lawrence J (2008) Migrants and tuberculosis: analysing epidemiological data with ethnography. Aust N Z J Public Health 32 (2): 142-9.

63. European Centre for Disease Prevention and Control (ECDC) (2009) Migrant health: Background note to the ‘ECDC Report on migration and infectious diseases in the EU’. Stockholm: ECDC.

64. Valin N, Antoun F, Chouaïd C, Renard M, Dautzenberg B, et al. (2005) Outbreak of tuberculosis in a migrants’ shelter, Paris, France, 2002. Int J Tuberc Lung Dis 9 (5): 528–33.

# Figure legends

Figure 1. The study selection process

# Tables

Table 1. The database searches

| **Databases** | **Search terms** | **Results** |
| --- | --- | --- |
| **Medline (Pubmed)** | **#1** "Tuberculosis"[Mesh] OR tb OR tuberculosis. **#2** "Transients and Migrants"[Mesh] OR "Emigration and Immigration"[Mesh] OR "Refugees"[Mesh] OR immigrant* OR migrant* OR migrat* OR refugee* OR foreign. **#3** "Qualitative Research"[Mesh] OR "Anthropology"[Mesh] OR "Ethnology"[Mesh] OR qualitative OR "Behavior"[Mesh]. **#1 AND #2 AND #3** | **559** |
| **Scopus** | TITLE-ABS-KEY. **#1** tb OR tuberculosis. **#2** transient* OR migrant* OR migrat* OR emigration OR immigration OR refugee OR refugees OR foreign. **#3** qualitative OR anthropology OR ethnology OR behavior. **#1 AND #2 AND #3.** | **544** |
| **JSTOR** | **Limits:** Item type: Article; Date range from 1995 to 2011; (Only accessible content via the University of Granada). **#1** tb OR tuberculosis. **#2** transient* OR migrant* OR migrat* OR emigration OR immigration OR refugee OR refugees OR foreign. **#3** qualitative OR anthropology OR ethnology OR behavior. **#1 AND #2 AND #3.** | **1285** |
| **Embase** | **#1** tb OR tuberculosis. **#2** transient* OR migrant* OR migrat* OR emigration OR immigration OR refugee OR refugees OR foreign. **#3** qualitative OR anthropology OR ethnology OR behavior. **#1 AND #2 AND #3** | **752** |

Table 2. Details of the included studies

| **#** | **Country** | **Authors** | **Methods** | **Participants and other details** |
| --- | --- | --- | --- | --- |
| 1 | US | AhChing et al, 2001 [23]. | Focus group. | 1 with 8 Samoan immigrants in Hawaii, TB patients or with latent TB, identified in a health centre. |
| 2 | US | Ailinger et al, 1997 [31]. | Individual interviews. | 65 Latin American immigrants (mainly from El Salvador), ill with TB in treatment. Identified through clinics and screening programmes. |
| 3 | US | Ho, 2003 [42]. | Individual interviews. | 60 Chinese immigrants ill with TB, in treatment. Identified in clinics or DOT program. |
| 4 | US | Ho, 2004 [4]. | Case study. | 1 female Chinese immigrant, TB patient. |
|  |  |  | Participant observation, structured questionnaires, illness narratives, reviews of medical records and analysis of epidemiological data. | Five groups of informants are included: public health workers, Chinatown biomedical doctors, Chinatown’s practitioners of traditional Chinese medicine, Chinese laborers and Chinese tuberculosis patients. |
| 5 | US | Houston et al, 2002 [35]. | Individual interviews. | 14 Vietnamese immigrants with latent TB, ill with TB (in treatment or not), or with TB in the past; key informants. Identified through health services and organizations. (and 18 follow-up telephone interviews) |
|  |  |  | Focus groups. | 8 with Vietnamese immigrants with latent TB, ill with TB (in treatment or not), or with TB in the past and key informants. Identified through health services and organizations (53 participants). |
|  |  |  | Community surveys. | 510 Vietmanese immigrants. |
| 6 | US | Ito, 1999 [45]. | Individual interviews. | 24 Vietnamese refugees ill with TB and community members, and staff from the clinic. Identified in the social health department. |
|  |  |  | Focus groups. | 1 with 12 Vietnamese refugees. |
|  |  |  | Participant observation. | In the main county public health clinic. |
| 7 | US | Joseph et al, 2008 [36]. | Individual interviews. | 50 Mexican immigrants identified in clinics after TB screening and adjacent communities, healthy individuals, latent TB and TB patients. |
| 8 | US | McEwen, 2005 [38]. | Individual interviews. | 9 Mexican immigrants diagnosed with latent TB and 5 of their partners. Identified in a TB clinic. |
|  |  |  | Participant observation, demographic questionnaire and review of medical records. | In participants’ homes, TB clinics and various health care settings on both sides of the border. |
| 9 | US | McEwen et al, 2007 [39]. | Individual interviews. | 8 female immigrants from Mexico with latent TB in risk of therapeutic failure. |
|  |  |  | Participant observation. | In the TB clinic and in the participants’ homes. |
| 10 | US | Poss, 1998 [30]. | Individual interviews. | 18 healthy Mexican farm immigrants (participants in a TB education program), identified in their household. Other 8 participated informally. |
| 11 | US | Wieland et al, 2012 [21]. | Focus groups. | 6 with healthy immigrants and refugees from Latin America, Africa and Asia, identified in an education centre (54 participants). 4 with staff from the education centre (29 participants, 10 of which immigrant). |
| 12 | US | Wyss et al, 2006 [47]. | Individual interviews. | 28 Latin American farm immigrants with latent TB identified after diagnosis. |
|  |  |  | Ethnographic observation. | In four commercial farms in Midwestern United States. |
| 13 | US | Yamada et al, 1999 [28]. | Focus groups. | 4 with Filipino immigrants, TB patients and healthy, identified from community health centres (36 participants). |
| 14 | New Zealand | Coreil et al, 2004 [34]. | Focus groups. | 5 with Haitian immigrants with HIV and TB, identified through the local health department (unknown exact number of participants). |
| 15 | New Zealand | Ng Shiu et al, 2008 [41]. | Individual interviews. | 11 Samoan (and Pacific Island) immigrants, TB patients identified in health centres. |
|  |  |  | Focus groups. | 2 with 12 Samoan community members who were not TB patients nor related to any TB patients. |
|  |  |  | Informal discussions. | With health professionals. |
| 16 | New Zealand | Van der Oest, 2005 [40]. | Individual interviews. | 7 representatives from seven main minority populations. Identified through a list of contacts. From Cambodia, China, Philippines, Samoa, Somalia Asia and Somalia, as well as Cook Islands and the Maori community. |
| 17 | UK | Brewin et al, 2006 [29]. | Individual interviews. | 53 African, Asian, Latin American and European immigrants identified in a social services centre, a clinic and health centre from TB screening. |
|  |  |  | Focus groups. | 1 with 4 African patients. |
| 18 | UK | Johnson, 2006 [25]. | Focus groups. | 9 with immigrants mainly from Vietnam, Somalia, China, Zimbabwe, and other African or Asian countries, identified in health services and organizations. |
|  |  |  | Individual interviews. | 1 of the groups had individual interviews instead focus group discussion (combining focus groups and individual interviews, there were 67 participants). |
| 19 | UK | Nnoaham et al, 2006 [33]. | Individual interviews. | 16 African immigrants, attending a clinic for TB treatment. |
| 20 | Canada | Bender et al, 2010 [43]. | Individual interviews. | 7 patients from TB centres and social services, mostly immigrants from diverse origins, and 9 Canadian female nurses. |
|  |  |  | Observation. | Observation of the visits between 9 nurses and 24 clients. |
| 21 | Canada | Gibson et al, 2005 [37]. | Individual interviews. | 133 immigrants from Asia and Eastern Europe identified from a TB clinic and aboriginals identified in the health centre. In treatment, prophylaxis, refusing prophylaxis or with personal or family history of TB. |
| 22 | China | Long et al, 2008 [44]. | Individual interviews. | 20 rural-to-urban migrant and permanent resident TB suspects identified from a cohort study, 17 TB patients and 23 key informants. |
|  |  |  | Focus group. | 12 with rural-to-urban migrants in the general population (unknown number of participants). |
|  |  |  | Prospective cohort study. | Adult suspect TB patients, migrant and urban residents. |
| 23 | China | Wei et al, 2009 [46]. | Individual interviews. | 34 rural-to-urban migrant TB patients, identified from a TB program. |
| 24 | Kazakhstan | Huffman et al, 2012 [22]. | Individual interviews. | 10 Uzbek migrant TB patients (identified from patients’ lists) and 18 health workers. |
|  |  |  | Focus groups. | 8 with Uzbek labor migrants and 4 with returned migrants in Uzbekistan. |
| 25 | Nepal | Kirwan et al, 2009 [32]. | Individual interviews. | 14 male rural-to-urban migrant TB patients, identified by health personnel in clinics. |
| 26 | Norway | Sagbakken et al, 2010 [26]. | Individual interviews. | 22 immigrant TB patients from Somalia and Ethiopia. Identified through primary health centres and hospitals. |
| 27 | Oman | AlManiri et al, 2010 [48]. | Individual interviews. | 17 health care providers, identified through direct referrals, with TB experience. The immigrant community was mainly from the Indian Subcontinent. |
| 28 | Spain | Blasco et al, 2010 [24]. | Individual interviews. | 45 immigrants ill with TB currently or previously, from various countries. Identified through the health services. |
| 29 | Sweden | Kulane et al, 2010 [49]. | Focus groups. | 5 with 34 Somali immigrants, TB patients. Men and women from social centres, mosques and community organizations. |
| 30 | Switzerland | Tardin et al, 2009 [27]. | Individual interviews. | 3, one immigrant TB patient, an interpreter and a cultural mediator. |
|  |  |  | Genotyping, contact tracing and literature review. |  |

**Supporting Information**
